# Metabolomic and Transcriptomic Analyses Reveal the Factors Underlying Mature Fruit Pericarp Color Variations in the ‘Xinli No. 7’ Pear (*Pyrus sinkiangensis*)

**DOI:** 10.3390/metabo15020081

**Published:** 2025-01-30

**Authors:** Yi Wang, Can Lu, Pan Yan, Shijie An, Ling Ma, Qiangqing Zheng, Yonghui Deng, Qiling Chen

**Affiliations:** 1 Xinjiang Production and Construction Corps, Key Laboratory of Korla Fragrant Pear Germplasm Innovation and Quality Improvement and Efficiency Increment, Tiemenguan Experimental Station, Xinjiang Academy of Agricultural and Reclamation Sciences, Tiemenguan 841000, China; wangyi@zhku.edu.cn (Y.W.); yanpan11235@163.com (P.Y.); asjzky@163.com (S.A.); 13209911390@163.com (L.M.); zhengqq369@163.com (Q.Z.); 2 College of Horticulture and Landscape Architecture, Zhongkai University of Agriculture and Engineering, Guangzhou 510225, China; 15602366712@163.com

**Keywords:** *Pyrus sinkiangensis*, fruit pericarp, peel color, polyphenol compounds, metabolomics, transcriptome

## Abstract

**Background/Objectives**: The ‘Xinli No. 7’ pear is a new pear variety with the advantages of early ripening, high quality, high storage resistance, and a long shelf life. Peel color is an important appearance-related trait and an important indicator of fruit quality and commercial value. **Methods**: In this study, we investigated the polyphenol compound biosynthesis metabolic pathway in the fruit pericarp of ‘Xinli No. 7’ pear using metabolomic and transcriptomic approaches, and qRT–PCR was used for the relative expression analysis of 21 DEGs associated with flavonoid biosynthesis. **Results**: A total of 128 phenolic compounds were identified, along with 1850 differently expressed genes (DEGs) in peels of different colors. Caftaric acid, apigenin, astragalin, phlorizin, prunin, taxifolin, rutin, naringenin, and their derivatives were abundant in the peel of ‘Xinli No. 7’ pear. An integrated analysis of transcriptomic and metabolomic data revealed that one PGT1, one LAR, two ANS, three 4CL, one CHS, one DFR, and one CHI gene involved in flavonoid biosynthesis exhibited markedly different expression levels in the fruit pericarp of ‘Xinli No. 7’ pear. Metabolic profiling of pear skin led to the identification of polyphenol substances involved in the flavonoid biosynthetic process and revealed 16 flavonoid compounds with high accumulation in pear fruit with red skin (PR). Notably, *MYBs* (25), *bHLHs* (18), *WRKYs* (15), *NACs* (15), *ERFs* (15), and *MADs* (2) may also contribute to the accumulation of flavonoid metabolites and the biosynthesis of anthocyanins in the peel of ‘Xinli No. 7’. **Conclusions**: Therefore, our results demonstrate the key role of phenolic compounds and candidate transcription factors involved in the peel color formation of ‘Xinli No. 7’ pear fruit.

## 1. Introduction

The ‘Xinli No. 7’ pear (‘*Korla fragrant*’ × ‘*Zaosu*’ hybrid), referred to as ‘fragrant pear’, has strong flavors, thin skin, and crispy flesh. It is a popular sweet and delicious pear cultivar in Xinjiang, and its production is increasing across China [1,2]. The ‘Xinli No. 7’ pear is a new early ripening pear variety derived using ‘Korla fragrant pear’ as the female parent and ‘Zaosu pear’ as the male parent through sexual cross-breeding at Tarim University in 1985. This variety has advantages such as early ripening, high quality, storage resistance, and a long shelf life [3,4]. During fruit ripening, the skin (pericarp) of the ‘Xinli No. 7’ pear is very smooth, with small inconspicuous lenticels, and is usually partially reddish [1,3]. Peel color is an important appearance characteristic and an important factor affecting fruit quality and commercial value. Red-peeled fruit is not only visually attractive but also has antioxidant effects on functional pigment components [4,5]. Red pears are more popular among consumers and are therefore more expensive [6,7]. Previous studies have investigated the anthocyanin content, pigment composition, and differentially expressed genes of green-skinned and red-skinned pear pericarp from various perspectives [4,5,6]. These studies have concluded that the red color of the pericarp is primarily due to differences in anthocyanoside content [4,5,6,7].

Compared with traditional green-, yellow-, and brown-peeled pear resources, red-peeled pear resources, which are deeply loved by consumers, are scarce and nutritious [8]. The formation of color in plant organs by MYB transcription factors is very important [9,10]. In previous studies in which green pears and red mutant pears were used as experimental materials, it was speculated that the R2R3-type MYB transcription factor gene *PcMYB10* was involved in anthocyanin synthesis during the formation of red peel [11]. Feng et al. [12] cloned and overexpressed *PcMYB10* in red pears to verify the function of this gene in regulating anthocyanin synthesis. Wang Zhimin [13,14], via Agrobacterium-mediated transient expression and stable expression, confirmed that three R2R3-type MYB transcription factor genes control the formation of red peel in the ’Zaosu’ pear. Specifically, *PbMYB10* promotes the expression of the anthocyanin synthesis structural gene *PbDFR, PbMYB9* promotes the expression of the *PbANR* and *PbUFGT1* genes, and *PbMYB3* promotes the expression of the *PbUFGT1* and *PbFLS* genes [15].

Flavonoids belong to a group of natural polyphenol compounds that are abundant in leaves, fruits, flowers, grains, and tea [16,17]. Although the flavonoid biosynthetic pathway is well characterized in other fruit colors, the molecular mechanisms that regulate red pericarp formation and pigment biosynthesis in the fruit pericarp of the ‘Xinli No. 7’ pear are still unclear. In this study, we investigated the phenolic compound biosynthesis metabolic pathway in the fruit pericarp of the ‘Xinli No. 7’ pear via metabolomic and transcriptomic methods. The aim of this study was to reveal the quantitative and qualitative differences in polyphenol substances and to analyze the differentially expressed genes (DEGs) and differentially abundant metabolites involved in pigment formation via bioinformatics approaches. The results provide not only target genes but also valuable information for the molecular breeding of pigment biosynthesis in the fruit pericarp of the ‘Xinli No. 7’ pear.

## 2. Materials and Methods

### 2.1. Plant Materials

**Figure 1:**
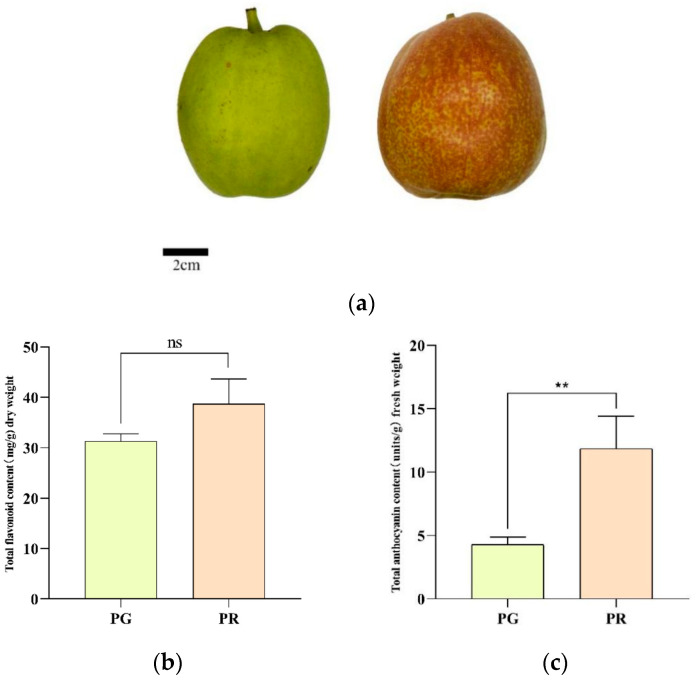
Determination of physiological characteristics of the fruit pericarp of ‘Xinli No. 7’ pear. (**a**) Phenotypes of ‘Xinli No. 7’ pear with a green pericarp (PG) and a red pericarp (PR). (**b**) Total flavonoid contents of PG and PR. (**c**) Relative anthocyanin contents of PG and PR. Each value is expressed as the mean ± standard deviation (*n* = 3), and the ** indicate significance at the 0.05 level. “ns” indicate "not significant".

The pear cultivar ‘Xinli No. 7’ was grown at the Pear Germplasm Research Center at the Institute of Forestry and Gardening, Xinjiang Academy of Agricultural and Reclamation, China, where the experiment started in the spring of 2023. At the end of August that year, fruits with green skin (PG) or red skin (PR) (ripe, free of pests and diseases, and undamaged) were collected from the test plants (Figure 1a). The intact fruits were stored in foam boxes at 4 °C and transported to the laboratory. The pear fruits were then peeled, and the red pericarp and green pericarp were collected in sterile freezing tubes. The pear pericarps were immediately frozen in liquid nitrogen for storage at −80 °C for RNA and metabolite extraction. Three biological replicates of samples of different colors were used.

### 2.2. Measurement of Total Flavonoids and Anthocyanins in the Fruit Pericarp of the ‘Xinli No. 7’ Pear

The total flavonoid content in the fruit pericarp of the ‘Xinli No. 7’ pear was quantified through aluminum nitrate colorimetry, in accordance with the method described by Jiang et al. [18]. Briefly, 0.5 mL of crude fruit peel extract was combined with 5.5 mL of 50% ethanol and 1 mL of a 5% NaNO_2_ solution. Following a six-minute incubation period, 1 mL of a 10% Al(NO_3_)_3_ solution was added, and the mixture was incubated for an additional six minutes. Subsequently, 10 mL of 4% NaOH solution and 7 mL of 50% ethanol were added to achieve a final volume of 25 mL, and the absorbance was measured at 506 nm using an ultraviolet-visible (UV) spectrophotometer (V-5100B, Metash, Shanghai, China).

The method for extracting total anthocyanins from the fruit pericarp of the ‘Xinli No. 7’ pear, as described by Fu et al. [19], was adopted, and the extraction conditions were optimized. The fruit pericarp was ground into powder in liquid nitrogen. Approximately 0.20 g of the dry powder was added to 20 mL of a 95% (0.1 mol L^−1^ HCl) ethanol solution and agitated at 25 °C for 2 h. The solution was then placed in a 4°C refrigerator and kept in the dark for 24 h. Finally, the absorbance of the extract was measured at 520 nm, 620 nm, and 650 nm using a BioTek microplate reader (Gene Company Limited, South San Francisco, CA, USA). The total anthocyanin content was calculated using the following formula: A = (A530 − A620) − 0.1(A650 − A620), and the anthocyanin content (mmol/g FW) was calculated as A × V × 1000/489.72 M, where V represents the volume of the extract and M represents the weight of the dried powder. A 95% (0.1 mol L^−1^ HCl) ethanol solution was used as a blank control. Each sample was analyzed in triplicate.

### 2.3. Sample Preparation and Metabolite Extraction

The pear pericarp was vacuum freeze-dried and ground into powder using liquid nitrogen, and 60 mg of the fruit pericarp samples were weighed and added to 600 μL of water/methanol (V/V = 1:2, containing the internal standards of succinic acid-2,2,3,3-d4 and salicylic acid-d4) and 400 μL of chloroform. This mixture was ground for 2 min with a grinding machine at 60 Hz. Ultrasonic extraction was then performed in an ice water bath for 20 min, followed by centrifugation for 10 min (4 °C, 13,000 rpm), and 200 μL of the supernatant was loaded into a new centrifuge tube. A total of 400 μL of water/methanol (V:V = 1:2, containing the internal standards of chloroform acid-2,2,3,3-d4 and salicylic acid-d4) was added to the residue, which was vortexed for 1 min, and ultrasonic extraction was performed for 20 min. The solution was centrifuged for 10 min (4 °C, 13,000 rpm), and 200 μL of supernatant was combined with the previous 200 μL of supernatant, resulting in a total volume of 400 μL. This 400 μL of supernatant was evaporated to dryness and then redissolved in 200 μL of water/methanol (V/V = 18:7, containing the internal standard of L-2-chlorophenylalanine), vortexed for 30 S, ultrasonicated for 2 min, and allowed to stand for 2 h at −20°C. Then, the supernatant was centrifuged for 10 min (4 °C, 13,000 rpm), and 150 μL of supernatant was transferred to a brown LC injection bottle and stored at −80 °C until analysis. All extraction reagents were pre-cooled at −20 °C before use.

### 2.4. Chromatography and Mass Spectrometry Acquisition Conditions

The samples were analyzed using high-performance liquid chromatography–electrospray ionization–tandem mass spectrometry (UPLC-ESI-MS/MS) under the following liquid phase conditions: column: Waters UPLC HSS T3 (100 × 2.1 mm, 1.8 μm); mobile phases: A 0.01% formic acid-water solution and B 0.04% acetonitrile solution; elution gradient: at 0 min, 5% B-phase; the B-phase proportion increased linearly to 95% in 11 min, was maintained at 95% for 1 min, decreased to 5% between 10.00 and 11.10 min, and then equilibrated at 5% until 14 min. The B-phase ratio decreased to 5% and equilibrated at 5% for 14 min; column temperature: 40 °C; flow rate: 0.35 mL/min; injection volume: 5 μL.

An electrospray ionization (ESI) source was detected using an AB4500Q TRAP UPLC/MS/MS system (equipped with an ESI Turbo Ion Spray interface), which was controlled by Analyst 1.6.3 software (AB Sciex). The ESI source operating parameters were as follows: ion source: turbo spray; source temperature: 500 °C; ion spray voltage (IS): 5500 V (positive ion mode)/−4500 V (negative ion mode); ion source gas I (GS I), gas II (GS II), and curtain gas (CUR): 60, 50, and 35.0 psi, respectively; and the parameter setting was high.

### 2.5. Qualitative and Quantitative Metabolite Analysis

The raw data were qualitatively analyzed using the Human Metabolome Database (HMDB), Lipidmaps (v2.3), and the METLIN database. Metabolite quantification was performed using triple quadrupole mass spectrometry in selected reaction monitoring (SRM) mode. Mass spectrometry data from different samples were obtained, peak area integration was performed for all peaks, and integration correction was performed for the peaks of the same substance across different samples.

### 2.6. Screening and Analysis of Differentially Abundant Metabolites

The mass spectrometry data were processed using Analyst 1.6.3 software (AB SCIEX, Toronto, ON, Canada). Multivariate statistical analysis was used to perform principal component analysis (PCA), hierarchical cluster analysis (HCA), and orthogonal partial least squares discriminant analysis (OPLS-DA) on the samples to further demonstrate the differences between the subgroups. PCA was performed using the built-in statistical prcomp function of R software 4.3.0 (www.r-project.org/, accessed on 17 October 2024), and the prcomp function parameter scale was set to “true” to indicate normalization of the data. Metabolite content data were normalized using UV normalization, and the normalized data were log_2_-transformed for further analysis. The heatmap was generated using R software and analyzed using the MetaboAnalystR package’s OPLSR.Anal function in R software. A selection of differentially abundant metabolites was performed by adopting a fusion of *p* value, fold change, and variable importance in projection (VIP) values from the OPLS-DA model, and the criteria for screening were a fold change ≥ 2, a fold change ≤ 0.5, *p* < 0.05, and a VIP > 1. The Kyoto Encyclopedia of Genes and Genomes (KEGG) database (https://www.genome.jp/kegg/, accessed on 17 October 2024) was used to annotate differentially abundant metabolites and analyze metabolic pathways.

### 2.7. RNA Isolation and Library Preparation

Total RNA was extracted using TRIzol reagent (Invitrogen, Carlsbad, CA, USA) according to the method described by Jiang et al. [18]. Briefly, a NanoDrop 2000 spectrophotometer (Thermo Scientific, Waltham, MA, USA, accessed on 16 September 2023) was used to measure RNA purity and concentration. The integrity of the total RNA from the fruit pericarp was assessed using an Agilent 2100 Bioanalyzer (Agilent Technologies, Santa Clara, CA, USA). The VAHTS Universal V6 RNA-seq Library Preparation Kit was subsequently used to construct the libraries according to the methods of Jiang et al. [18] and Fu et al. [19]. Transcriptome sequencing and analysis were conducted by Shanghai OE Biotech Co., Ltd., China (Shanghai, China).

### 2.8. RNA Sequencing and Differentially Expressed Gene Analysis

Clean reads were obtained on the basis of previous methods [18,19]. Then, approximately 273,180,000 clean reads for each sample were retained for subsequent analyses. The clean reads were mapped to the reference genome (*Pyrus_bretschneideri*_v2.0) using HISAT22. The FPKM3 value of each gene was calculated, and the read count of each gene was obtained via HTSeq-count4. PCA was performed using R (v 3.2.0) to evaluate the biological duplication of samples. Differential expression analysis was performed via DESeq25. A Q value < 0.05 and a fold change ≥ 1.5 or a fold change ≤ 0.67 were set as the thresholds for significant differential gene expression. Hierarchical cluster analysis of DEGs was performed using R (v 3.2.0) to demonstrate the expression patterns of genes across different groups and samples. Gene function was annotated using the following databases: the Kyoto Encyclopedia of Genes and Genomes (KEGG) (https://www.genome.jp/kegg, accessed on 27 September 2024), the Gene Ontology (GO) database (https://www.geneontology.org, accessed on 25 September 2024), and the Pfam database (version 33.0). For the analysis, a predefined gene set was used, and the genes were ranked according to the degree of differential expression in the two types of samples. Next, we tested whether the predefined gene set was enriched at the top or bottom of the ranking list.

### 2.9. Validation of DEGs by Quantitative Real-Time PCR

The expression patterns of 21 DEGs involved in flavonoid biosynthesis were validated via qRT–PCR (Table S1 displays gene-specific primers). The actin gene (internal control) was used as an internal control for normalization of gene expression. Total RNA from the *P. bretschneideri Rehd* pericarp was extracted using the same methods described above for RNA-Seq. RT–qPCR was conducted using RealUniversal Color PreMix (SYBR Green) according to the manufacturer’s instructions (Tiangen, Beijing, China). Quantitative real-time polymerase chain reaction (qRT–PCR) was conducted according to the approach described by Jiang et al. [18]. The comparative CT method (2^−ΔΔCT^ method) was subsequently used to quantify gene expression.

## 3. Results

### 3.1. Determination of Total Flavonoids and Anthocyanins in the Fruit Pericarp of the ‘Xinli No. 7’ Pear

To determine the physiological characteristics of the fruit pericarp of the ‘*Xinli No. 7*’ pear, the total flavonoid and anthocyanin contents were determined. The results revealed that the total flavonoid content of PR was approximately 38.69 mg/g dry weight, which was greater than the value of 31.27 mg/g dry weight observed for PG (Figure 1b), and the relative anthocyanin content of PR was 11.85 units/g fresh weight, which was significantly greater than the value of 4.29 units/g fresh weight for PG (Figure 1c).

### 3.2. Metabolomic Analysis and Metabolite Identification

**Figure 2:**
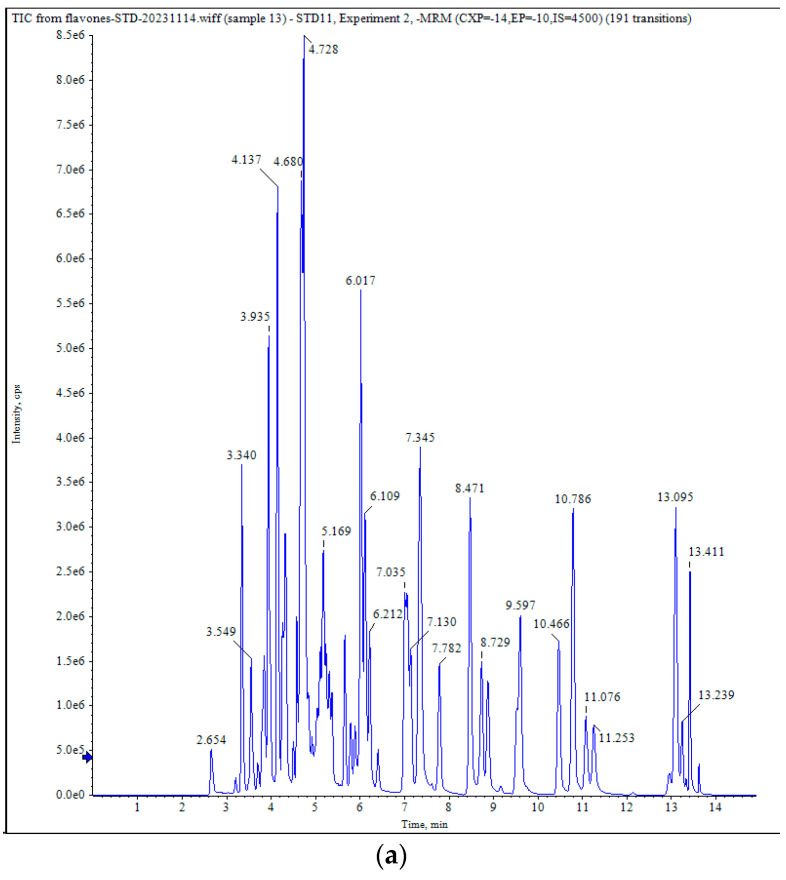

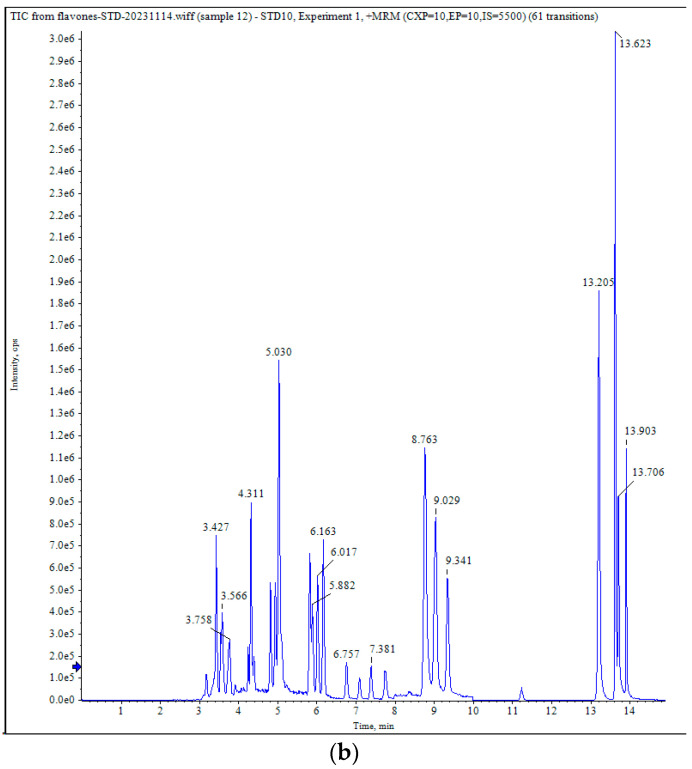
Detailed examination of the total ion flow diagram for metabolite detection in the fruit pericarp of ‘Xinli No. 7’ pear. (**a**) Negative ion mode; (**b**) Positive ion mode.

The mass spectrometry data were processed using Analyst 1.6.3 software, and the total ion currents (TICs) of the mixed quality control (QC) samples are shown in Figure 2. The horizontal coordinate depicts the retention time (min) of the metabolite assay, whereas the vertical coordinate represents the intensity of the detected ion currents (cps: counts per second). The MRM parameters for the analysis of metabolites are shown in Table S2. The results demonstrated that the TIC curves of the various QC samples exhibited overlapping patterns during the metabolite assay, with consistent retention times and peak intensities. This finding indicates that the experimental process was stable and that the assay results were reliable.

### 3.3. Profiling of Phenolic Compounds in the Fruit Pericarp of the ‘Xinli No. 7’ Pear

**Figure 3:**
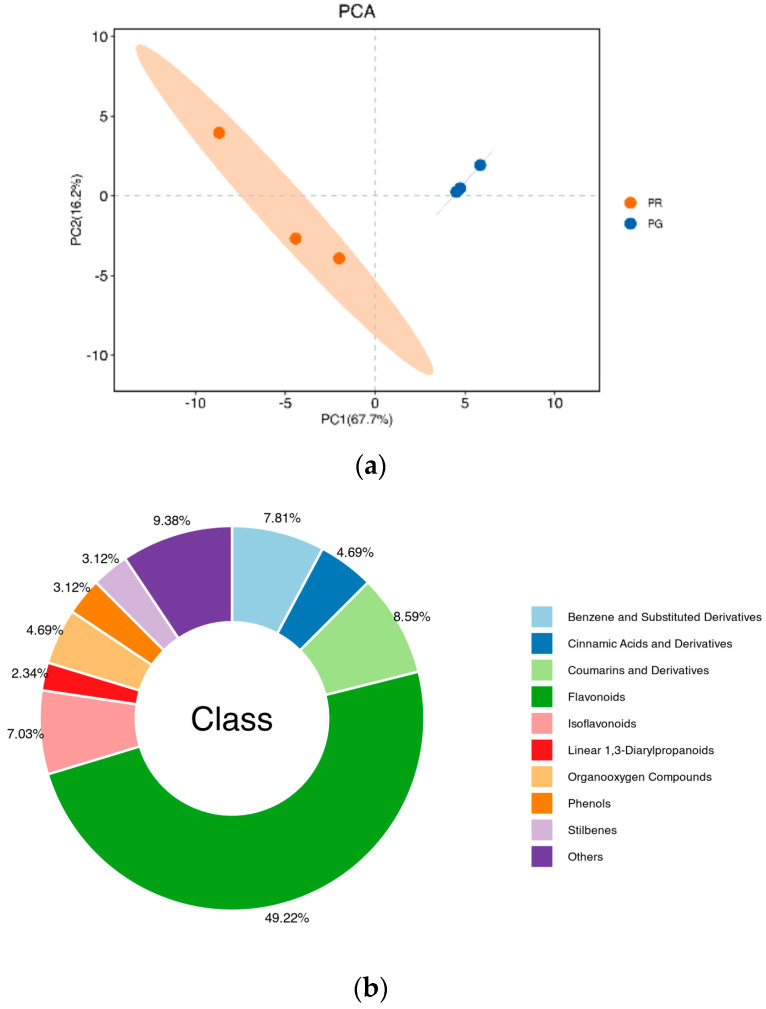
Analysis of phenolic metabolites in the fruit pericarp of ‘Xinli No. 7’ pear. (**a**) PCA clustering of the metabolome data. (**b**) Component analysis of the identified metabolites.

The results of PCA revealed a high degree of dispersion between the PR and PG groups, with obvious metabolic differences (Figure 3a). The principal component scores revealed that PC1 and PC2 explained 67.7% and 16.2% of the variability among the samples, respectively, and the total contribution rate reached 83.9% (Figure 3a). The results for the peel samples of the two colors were clearly separated and reproducible, and the replicates were closely clustered, indicating high reproducibility and scientific reliability of the data. In this work, a total of 128 phenolic compounds were identified in the fruit pericarp of the ‘Xinli No. 7’ pear (Table S3). The 128 phenolic compounds were classified into 10 categories, including 63 flavonoids (49.22%), 11 coumarins and derivatives (8.59%), 10 benzene and substituted derivatives (7.81%), 6 cinnamic acids and derivatives (4.69%), 6 organooxygen compounds (4.69%), 6 organooxygen compounds (4.69%), 4 stilbenes (3.12%), 4 phenols (3.12%), 4 stilbenes (3.12%), 3 linear 1,3-diarylpropanoids (2.34%), and 12 unclassified compounds (9.38%) (Figure 3b).

### 3.4. Analysis of Differentially Abundant Metabolites in the Fruit Pericarp of the ‘Xinli No. 7’ Pear

**Figure 4:**
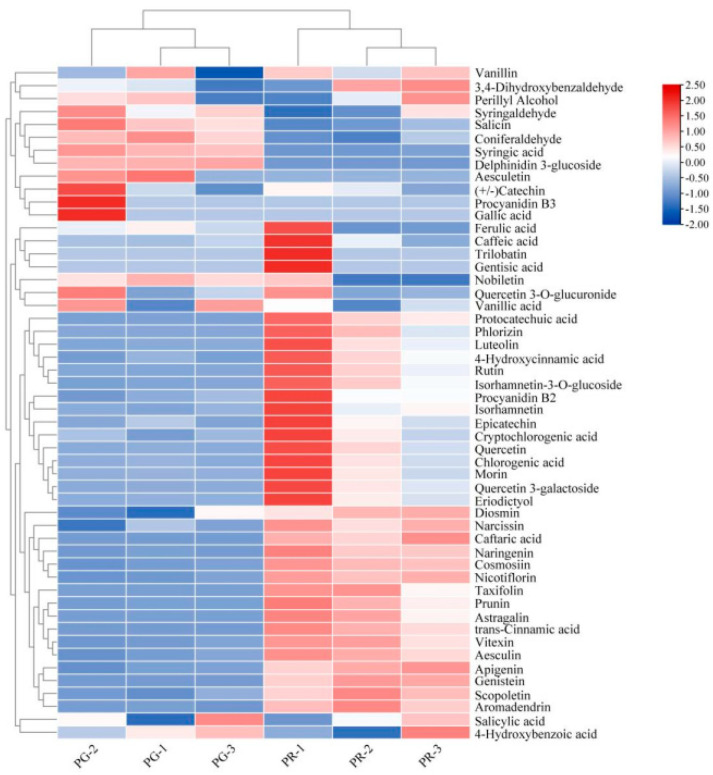
Analysis of phenolic compounds in the fruit pericarp of ‘Xinli No. 7’ pear. Cluster heatmap of differentially abundant phenolic compounds between the PR and PG groups. Each sample is represented by a column, and each metabolite is represented by a row. The abundance of each metabolite is represented with a different color. High-abundance metabolites are shown in red, whereas low-abundance metabolites are shown in blue.

A heatmap of the differentially abundant metabolites was generated using R software, following unit variance (UV) scaling, and a hierarchical cluster analysis (HCA) was performed on the accumulation patterns of the metabolites among the different samples (Figure 4). According to the identification criteria of a fold change ≥ 1.50 or ≤ 0.67 and VIP ≥ 1, 25 metabolites among the 128 phenolic compounds were identified as differentially abundant, including 36 enriched metabolites and 17 depleted metabolites (Figure 4, Table S3). In total, 63 flavonoid metabolites, such as nicotiflorin, cosmosiin, apigenin, aromadendrin, naringenin, and vitexin, were identified in the fruit pericarp of the ‘Xinli No. 7’ pear (Table S3). Among the 53 differentially abundant metabolites, thirteen were related to flavonoids, with high contents of astragalin, taxifolin, isorhamnetin-3-O-glucoside, rutin, phlorizin, luteolin, and genistein found in the fruit pericarp of the ‘Xinli No. 7’ pear (Table 1). Naringenin, taxifolin, and trans-cinnamic acid may be responsible for the red pericarp color of the ‘Xinli No. 7’ pear.

### 3.5. Differentially Expressed Genes in the Fruit Pericarp of the ‘Xinli No. 7’ Pear

**Figure 5:**
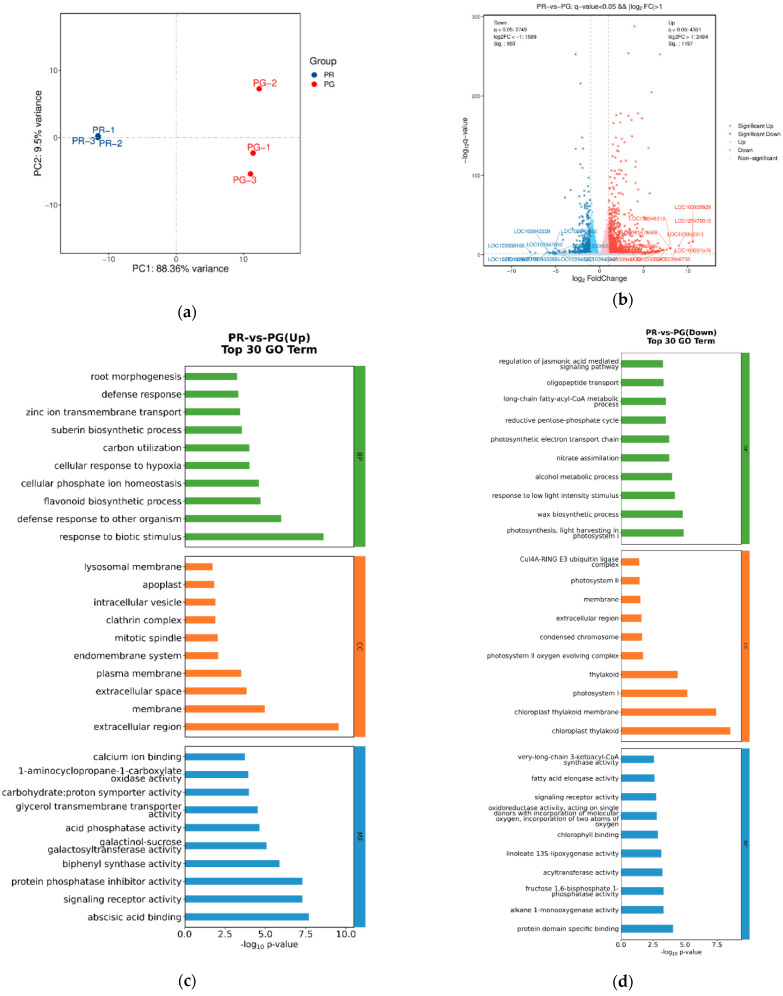

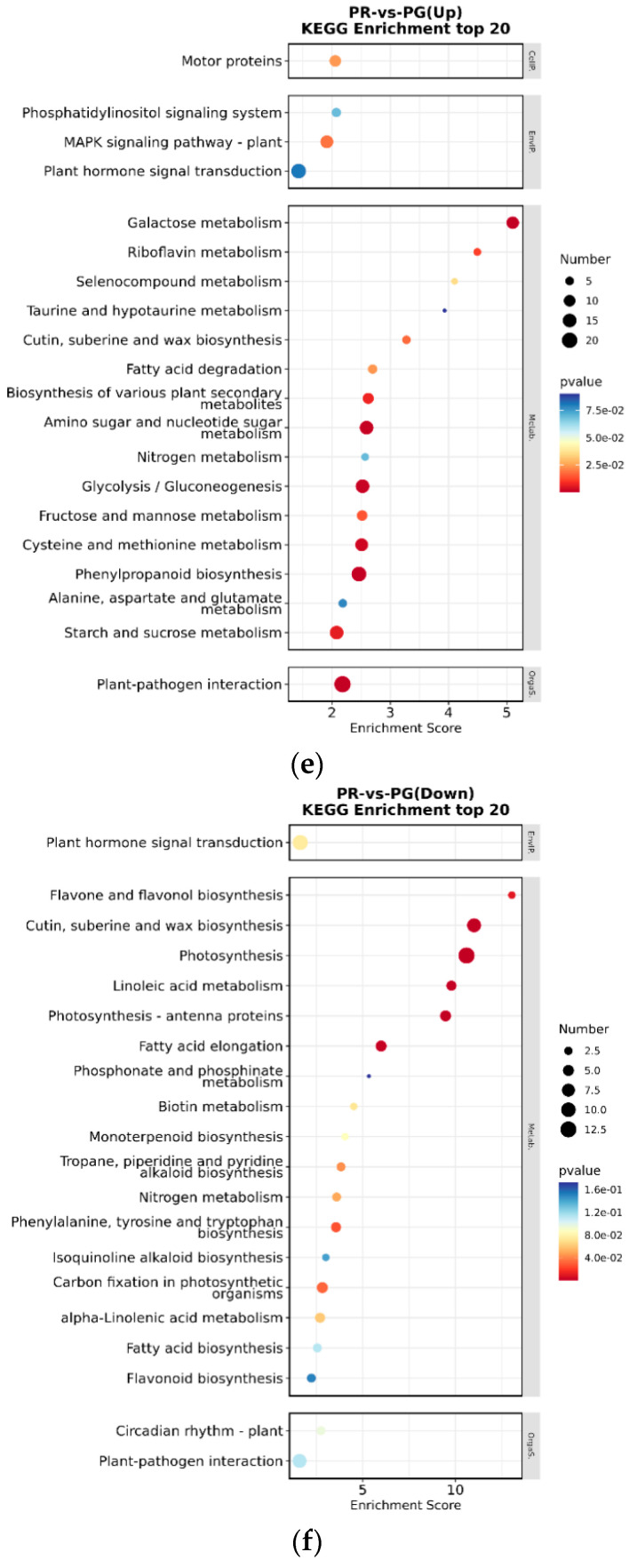
Analysis of differentially expressed genes in the fruit pericarp of ‘Xinli No. 7’ pear. (**a**) PCA of the PR and PG samples. (**b**) Volcano plot of the genes differentially expressed between the green pericarp and red pericarp. (**c**,**d**) GO functional classification of DEGs. (**e**,**f**) KEGG pathway enrichment of the DEGs. The significantly differentially expressed genes were enriched in several metabolic processes, including phenylpropanoid pathways.

To understand the molecular basis of peel coloration in pear fruit, transcriptomes were analyzed to identify DEGs in the pericarp of the ‘Xinli No. 7’ pear. A total of 273.18 million clean reads were produced from the fruit pericarp of ‘Xinli No. 7’. An overview of the sequencing and mapping results is shown in Table 2. An average of 45,529,529 reads were obtained, with a Q30 percentage of no less than 94.48% in each sample. These clean reads were further assembled into 31,689 unigenes with a mean length of 1268.19 bp using Trinity software. Among the 316,898 assembled unigenes, 23,318 (73.58%) were functionally annotated via the abovementioned databases. The results of PCA revealed a high degree of dispersion between the PR and PG groups, with obvious differences in gene expression (Figure 5a). Using the filter criteria of a fold change ≥ 2.0 or a fold change ≤ 0.5 and an FDR < 0.05, 1850 DEGs were identified in the fruit pericarp, including 1197 upregulated genes and 653 downregulated genes (Figure 5b, Table S4). In the GO enrichment analysis of the DEGs, 1525 out of 1850 were found to be involved in three major GO categories, i.e., biological process, cellular component, and molecular function (Figure 5c,d). As illustrated in Figure 5c, 1197 upregulated genes related to GO terms were enriched, including those associated with flavonoid biosynthetic processes (GO:0009813), response to biotic stimulus (GO:0006952), and abscisic acid binding (GO:0010427). However, as illustrated in Figure 5d, 653 downregulated genes related to GO terms were enriched in chloroplast thylakoid (GO:0009534), photosynthesis, light harvesting in photosystem I (GO:0009768), and protein domain-specific binding (GO:0019904).

To fully grasp the intricacies of molecular functions and gene information, we annotated a comprehensive set of 14,267 unigenes, which encompassed 1850 DEGs, using the KEGG database. These genes were meticulously organized into 102 distinct metabolic pathways. When a KEGG metabolic pathway enrichment analysis was conducted with a Q value threshold of less than 0.05, our findings revealed that the DEGs were significantly enriched in various metabolic processes. Specifically, these pathways included phenylpropanoid biosynthesis, glycolysis/gluconeogenesis, flavonoid biosynthesis, and starch and sucrose metabolism pathways (Figure 5e,f).

### 3.6. Combined Transcriptomic and Metabolomic Analysis Revealed the Biosynthesis of Phenolic Compounds in the Fruit Pericarp of the ‘Xinli No. 7’ Pear

**Figure 6:**
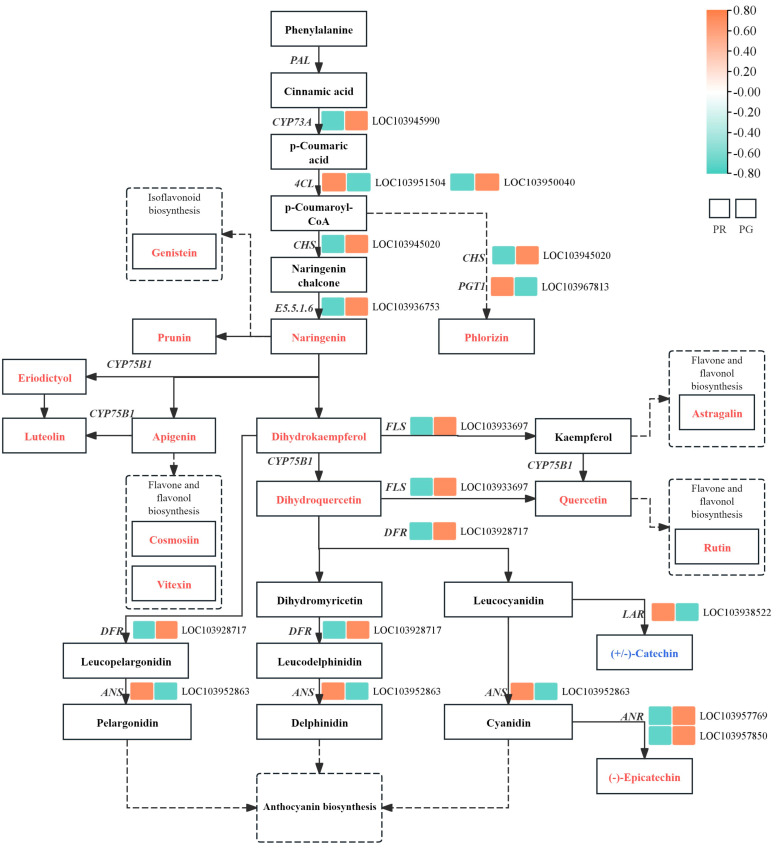
Biosynthetic pathway for flavonoids in the peel of ‘Xinli No. 7’. The substances noted in the boxes are metabolites, with red indicating a greater content of the metabolite in PR than in PG and blue indicating the opposite trend. The heatmap shows the changes in transcripts involved in flavonoid biosynthesis. The upregulated genes are shown in orange-red, and the downregulated genes are shown in green.

Through a combined analysis of the DEGs and metabolite data, we investigated the phenolic biosynthesis pathway in the peel of the ‘Xinli No. 7’ pear. The results demonstrated that many flavonoids were detected in the pear fruit peel. The identified flavonoids were involved in the flavonoid biosynthesis pathway (Figure 6). In this pathway, we found that genistein, prunin, naringenin, phlorizin, rutin, quercetin, taxifolin, and luteolin were more abundant in PR than in PG. Furthermore, an examination of the genes related to flavonoid metabolism revealed significant variations in the expression levels of 15 pivotal unigenes, with 4 exhibiting upregulation and 11 demonstrating downregulation in PR compared with PG. Among the DEGs, one *PGT1*, one *4CL*, one *LAR*, and one *ANS* gene were upregulated 1.53- to 116.09-fold (fold change), whereas one *CYP73A*, two *4CL*, one *CHS*, one *DFR*, one *ANS*, and one *CHI* gene were downregulated 0.03- to 0.54-fold (Figure 6). These DEGs were associated with the biosynthesis of flavonoids in the peel of the ‘Xinli No. 7’ pear.

### 3.7. Transcription Factors Involved in Flavonoid Biosynthesis in the Fruit Pericarp of the ‘Xinli No. 7’ Pear

**Figure 7:**
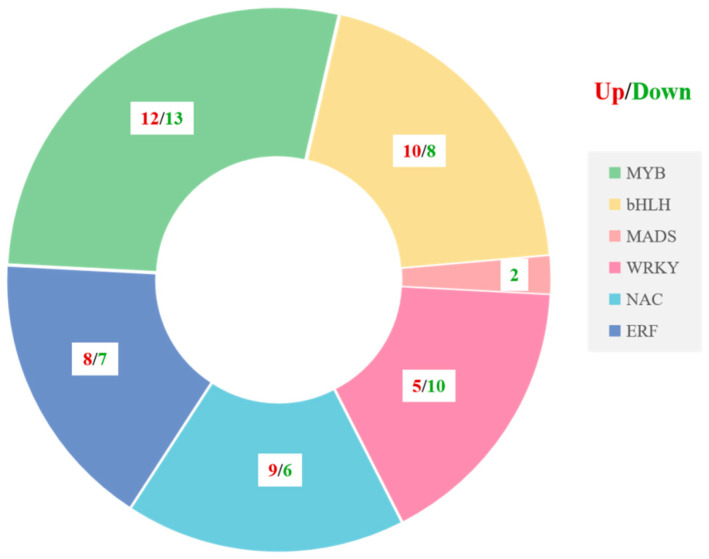
Transcription factors involved in flavonoid biosynthesis in the fruit peel of the ‘Xinli No. 7’ pear.

Transcription factors play a crucial role in the intricate processes of flavonoid biosynthesis by meticulously regulating gene expression within plants. We identified ninety transcription factors linked to flavonoid biosynthesis with varying expression levels (Figure 7). Among the transcription factors discovered within peels of ‘Xinli No. 7’, MYBs (25) and bHLHs (18) were the most prevalent, followed closely by WRKYs (15), NACs (15), ERFs (15), and MADs (2) (Figure 7). Notably, most bHLHs and NACs were upregulated. These transcription factors may contribute to the accumulation of flavonoid metabolites and the biosynthesis of anthocyanins in the peel of ‘Xinli No. 7’, thereby promoting red coloration in pear skin.

### 3.8. qRT–PCR Qualification and Analysis

**Figure 8:**
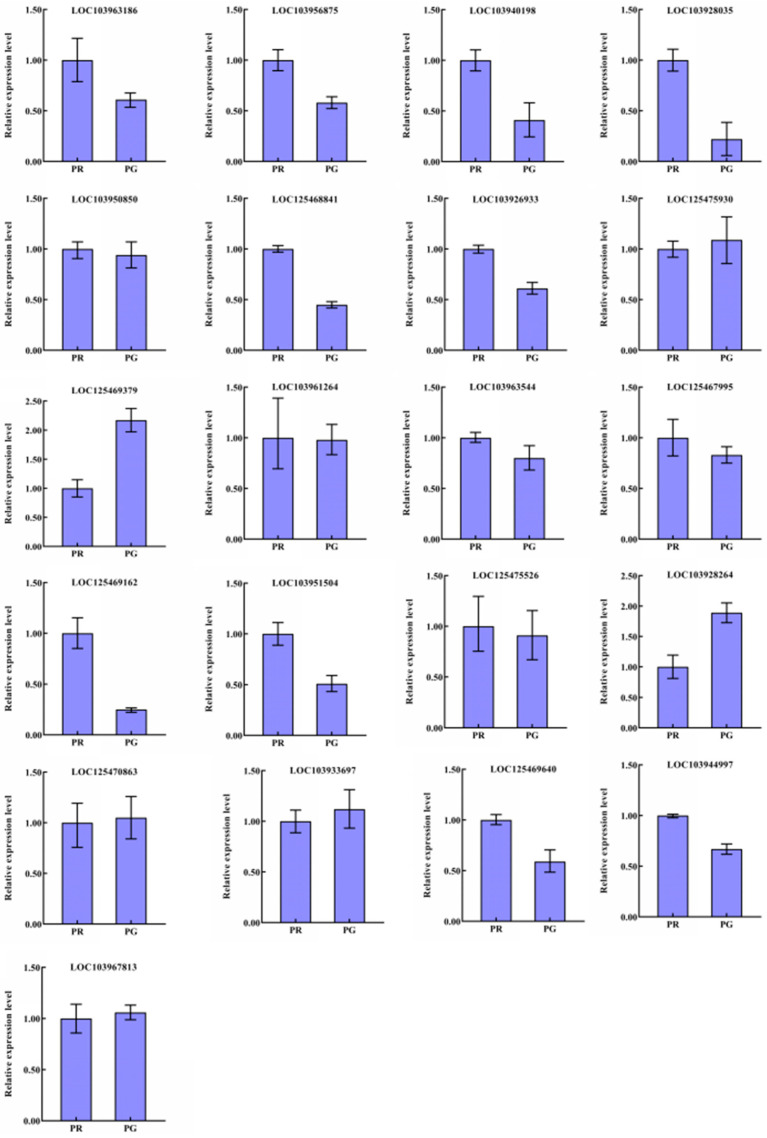
Relative expression profiles of 21 genes involved in flavonoid biosynthesis in the fruit peel of the ‘Xinli No. 7’ pear, determined via qRT–PCR analysis. The data are presented as means ± standard deviations (n = 3).

To validate the reliability of the transcriptome data fort the ‘Xinli No. 7’ pear acquired through RNA-seq, a set of 21 DEGs related to flavonoid biosynthesis was subjected to qRT–PCR expression analysis. The expression profiles of the 19 DEGs determined via qRT–PCR were highly consistent with those determined via RNA-seq (Figure 8), suggesting that the transcriptome-based analysis of the ‘Xinli No. 7’ pear was highly accurate and reliable.

## 4. Discussion

Peel color is an important appearance-related trait and an essential determinant of fruit quality, as well as an excellent indicator of fruit’s commercial value; it is also physiologically important for resistance to biotic and abiotic stresses during fruit development [20,21,22]. Red-peeled pears are more popular among consumers. The rapid accumulation of secondary metabolites, such as anthocyanin glycosides, during fruit development improves fruit coloration [23]. However, to our knowledge, the underlying regulatory network for fruit color in pears remains unknown. To investigate the molecular regulatory mechanisms for pear fruit coloration, transcriptomic and metabolomic studies were carried out in this study on pear fruit with differently colored peels. The combination of metabolomics and transcriptomics has been widely used to investigate metabolite biosynthesis and reveal the biosynthetic pathways of metabolites in plants. The red peel of ‘Xinli No. 7’ pears is not only visually appealing but also rich in functional pigment components with antioxidant effects. In the present study, the total flavonoid content extracted from the PR samples was significantly greater than that extracted from the PG samples (Figure 1). To elucidate the mechanisms of peel coloration in the fruit of ‘Xinli No. 7’, metabolomic and transcriptomic data were collected for the fruit pericarp of PR and PG. A total of 128 phenolic compounds were identified in the fruit pericarp of ‘Xinli No. 7’ pears via UPLC/ESI-Q TRAP-MS/MS. Among these metabolites, 53 were significantly differentially abundant between PR and PG, including 36 enriched metabolites and 17 depleted metabolites (Figure 3b). To date, few studies have conducted qualitative and quantitative research on the phenolic compounds in pear peels or their biosynthetic pathways. The most abundant phenolic compounds in the peel of Korla fragrant pears are flavonoids, with 63 flavonoids (49.22%) detected in this study (Table S3). According to our data, the backbone structure of most flavonoids in the peel of ‘Xinli No. 7’ pears includes apigenin, naringenin, rutin, taxifolin, phlorizin, quercetin, and catechin.

Phenolic compounds are secondary metabolites found in many plant foods and play important roles in fruit and leaf color formation, in addition to contributing to improvements in food quality and organoleptic properties [24,25]. Phenolic compounds can be categorized according to their structure into flavonoids, phenolic acids, and tannins [26]. In this study, transcriptomic analysis of the peel of the ‘Xinli No. 7’ pear revealed unigenes involved in the flavonoid biosynthesis process, and DEGs were identified. The expression levels of genes encoding PGT1, 4CL, LAR, and ANS involved in flavonoid biosynthesis were greater in PR than in PG (Figure 6), which resulted in a greater flavonoid content in PR. According to the metabolite and DEG data, we speculated that the *ANS* gene was upregulated 1.54-fold (fold change), and that high expression of the *ANS* gene may increase the accumulation of anthocyanin compounds in PR. A comprehensive analysis of transcriptomics and metabolomics can be an advantage in comprehending the biological functions of secondary metabolites in peel color formation. Jiang et al. demonstrated that high expression levels of the *CHS* gene increase the accumulation of flavonoids in red perilla compared with green perilla [18]. In the flavonoid biosynthesis pathway, the expression levels of genes encoding *ANS, PGT1, 4CL*, and *LAR* were greater in PR than in PG (Figure 6), which may have resulted in a greater flavonoid content in PR. *ANS* is an important dioxygenase in plants that is capable of catalyzing the conversion of leucoanthocyanidins to anthocyanins and the conversion of dihydroquercetin to quercetin [27,28]. The overexpression of *MaANS* in tobacco leads to an increase in the color intensity of corollas, which is achieved through an increase in anthocyanin accumulation [26]. Moreover, a previous study demonstrated that *PGT1, 4CL*, and *LAR* are the most important genes for flavonoid biosynthesis in fruits [29,30]. In this study, metabolic profiling of pear skin led to the identification of flavonoid metabolites involved in the flavonoid biosynthetic process and revealed 16 flavonoid compounds with high accumulation in the PR group. On the basis of the transcriptomic and metabolic analyses, we hypothesized that increased expression of the *ANS* gene and the high accumulation of these 16 flavonoid compounds enhanced the formation of peel color in red fruits.

The biosynthesis of flavonoids is a complex and delicate process in which transcription factors play crucial regulatory roles. The MYB-bHLH-WD40 complex, zinc fingers, MADS-box family members, NACs, and WRKY proteins have been recognized as significant contributors to the flavonoid biosynthetic pathway in plants [18,31,32,33]. The synthesis and accumulation of flavonoids in various parts of Arabidopsis seedlings, such as roots, stems, and leaves, are regulated by R2R3-MYB transcription factors [34]. The MYB12 transcription factor has been found to regulate flavonol accumulation by modulating the expression of key genes involved in flavonol biosynthesis [35]. Furthermore, the transcription factor WRKY23 from *Arabidopsis* governs flavonol biosynthesis and accumulation during root development [36]. Notably, the expression levels of *bHLH* transcription factors are significantly greater in perilla with red leaves than in perilla with green leaves [18,37]. Specifically, the bHLH factor from perilla, which modulates the expression of genes involved in the anthocyanin metabolic pathway, was detected exclusively in red perilla leaves but was absent in green leaves [38]. In the present study, we analyzed and counted transcriptome data, revealing that ninety pivotal transcription factors, including MYBs, ERFs, WRKYs, bHLHs, MADSs, and NACs, presented markedly distinct expression patterns (Figure 8). These differentially expressed transcription factors have emerged as potential regulators of flavonoid biosynthesis in the peel of ’Xinli No. 7’, potentially facilitating the development of red peel in pear fruit. Further work is being carried out to support this hypothesis and to investigate the possibility of spraying catalytically active compounds in relevant metabolic pathways, with the aim of promoting the red coloration of pear fruit.

## 5. Conclusions

In conclusion, integrated metabolic and transcriptomic analyses were performed to understand the potential regulatory pathways involved in peel color formation in pear fruit. The peel of ‘Xinli No. 7’ was employed as the test material for the first time, resulting in the identification of a total of 128 phenolic compounds, along with 1850 DEGs between differently colored peels. Caftaric acid, apigenin, astragalin, phlorizin, prunin, taxifolin, rutin, naringenin, and other phenolic compounds were abundant in the peel of ‘Xinli No. 7’. Integrated analysis of transcriptomic and metabolomic data revealed that one *PGT1*, one *LAR*, two *ANS*, three *4CL*, one *CHS*, one *DFR*, and one *CHI* gene involved in flavonoid biosynthesis exhibited markedly different expression levels in the fruit pericarp of the ‘Xinli No. 7’ pear. Metabolic profiling of pear skin identified flavonoid metabolites involved in the flavonoid biosynthetic process and revealed 16 flavonoid compounds with high accumulation in the PR group. Moreover, the transcription factors MYBs (25), bHLHs (18), WRKYs (15), NACs (15), ERFs (15), and MADs (2) may also be involved in regulating the accumulation of flavonoid metabolites and anthocyanin biosynthesis in the pear fruit pericarp. Our results provide new insights into candidate genes involved in the pathway of phenolic compound biosynthesis during pear peel color formation and their applications in breeding.

## Figures and Tables

**Table 1:** Partial list of differentially abundant phenolic compounds between the PR and PG groups.

Class	Metabolites	Regulation	Fold Change PR/PG	*p* Value
Benzene and substituted derivatives	Syringic acid	Down	0.22	0.0001
	Protocatechuic acid	Up	7.26	0.00875
Cinnamic acids and derivatives	Caftaric acid	Up	221.93	0.00058
Flavonoids	trans-Cinnamic acid	Up	34.97	0.00098
	4-Hydroxycinnamic acid	Up	7.42	0.02477
	Aesculin	Up	2.99	0.00085
	Scopoletin	Up	2.52	0.00131
	Delphinidin 3-glucoside	Down	0.00	1 × 10^−6^
	Nicotiflorin	Up	3.52	5.9 × 10^−5^
	Cosmosiin	Up	12.64	0.00014
	Apigenin	Up	7.20	0.00046
	Aromadendrin	Up	2.28	0.00066
	Naringenin	Up	7.13	0.00108
	Vitexin	Up	1.59	0.00112
	Prunin	Up	18.93	0.00406
	Astragalin	Up	81.92	0.00418
	Taxifolin	Up	47.57	0.00418
	Isorhamnetin-3-O-glucoside	Up	14.53	0.01939
	Rutin	Up	7.24	0.03038
	Phlorizin	Up	38.52	0.03367
	Luteolin	Up	16.95	0.04145
Isoflavonoids	Genistein	Up	80.83	0.00024
Organooxygen compounds	Salicin	Down	0.55	0.00576
Phenols	Coniferaldehyde	Down	0.55	0.00492
Unclassified	Narcissin	Up	1.49	0.00563

**Table 2:** Overview of the sequencing and mapping results.

Sample	Read Number	Base Number (G)	Q30 Percentage (%)	GC Content (%)	Mapped Reads	Mapped Ratio (%)
PG-1	48,149,428	7.29	94.58	45.95	45,533,514	94.57
PG-2	48,225,308	7.31	94.48	45.99	45,514,412	94.38
PG-3	43,309,128	6.55	94.58	46.01	40,960,764	94.58
PR-1	44,232,248	6.68	94.50	46.08	41,852,620	94.62
PR-2	42,864,618	6.48	94.56	46.09	40,573,299	94.65
PR-3	46,396,444	7.01	94.55	46.09	43,908,776	94.64
Average	45,529,529	6.89	94.54	46.04	43,057,231	

Q30 percentage: the proportion of nucleotides with a quality value of ≥30. GC content: the proportion of guanidine and cytosine nucleotides. Mapped ratio: the percentage of mapped reads in proportion to clean reads.

## Data Availability

All data generated or analyzed during this study are included in this published article (and its Supplementary Materials files).

## Supplementary Materials

The following supporting information can be downloaded at: https://www.mdpi.com/article/10.3390/metabo15020081/s1, Table S1: Primers Sequence; Table S2: The MRM parameters for analysis of metabolites in the fruit pericarp of ‘Xinli No.7’ pear; Table S3: Phenolic compounds identified in the fruit pericarp of ‘Xinli No.7’ pear; Table S4: 1850 differentially expressed genes in the fruit pericarp of ‘Xinli No.7’ pear.
